# Absence of association between whole blood viscosity and delirium after cardiac surgery: a case-controlled study

**DOI:** 10.1186/s13019-016-0517-9

**Published:** 2016-08-05

**Authors:** Shokoufeh CheheiliSobbi, Mark van den Boogaard, Arjen J. C. Slooter, Henry A. van Swieten, Linda Ceelen, Gheorghe Pop, Wilson F. Abdo, Peter Pickkers

**Affiliations:** 1 Department of Intensive Care Medicine, Radboudumc, Nijmegen, The Netherlands; 2 Department of Cardiothoracic Surgery, Radboudumc, Nijmegen, The Netherlands; 3 Department of Cardiology, Radboudumc, Nijmegen, The Netherlands; 4 Department of Intensive Care Medicine, University Medical Centre Utrecht, Utrecht, The Netherlands

**Keywords:** Cardiothoracic surgery, Delirium, Intensive care, Medicine, Whole blood viscosity

## Abstract

**Background:**

Delirium after cardiothoracic surgery is common and associated with impaired outcomes. Although several mechanisms have been proposed (including changes in cerebral perfusion), the pathophysiology of postoperative delirium remains unclear. Blood viscosity is related to cerebral perfusion and thereby might contribute to the development of delirium after cardiothoracic surgery. The aim of this study was to investigate whether whole blood viscosity differs between cardiothoracic surgery patients with and without delirium.

**Methods:**

In this observational study postoperative whole blood viscosity of patients that developed delirium (cases) were compared with non-delirious cardiothoracic surgery patients (controls). Cases were matched with the controls, yielding a 1:4 case–control study. Serial hematocrit, fibrinogen, and whole blood viscosity were determined pre-operatively and at each postoperative day. Delirium was assessed using the validated Confusion Assessment Method for the Intensive Care Unit or Delirium Screening Observation scale.

**Results:**

In total 80 cardiothoracic surgery patients were screened of whom 12 delirious and 48 matched non-delirious patients were included. No significant difference was found between both groups in fibrinogen (*p* = 0.36), hematocrit (*p* = 0.23) and the area under curve of the whole blood viscosity between shear rates 0.02 and 50 s^-1^ (*p* = 0.80) or between shear rates 0.02 and 5 s^-1^ (*p* = 0.78).

**Conclusion:**

In this case control study in cardiothoracic surgery patients changes in whole blood viscosity were not associated with the development of delirium.

## Background

Delirium is a serious neuropsychiatric disorder characterized by an acute onset of altered mental status, hallmarked by difficulty in sustaining attention with typically a fluctuating course [1]. Delirium occurs frequently in hospitalized patients, especially in Intensive Care Unit (ICU) patients [2]. In cardiothoracic surgery the incidence of delirium during the postoperative on the ICU is reported to be between 13 % and 42 % [3, 4]. Postoperative delirium in cardiothoracic surgery patients is associated with increased length of ICU and hospital stay, increased risk of sternal wound infection, unwanted removal of arterial/venous lines or epicardial electrodes, significantly impaired quality of life and higher long-term morbidity, mortality and healthcare costs [5, 6].

The pathophysiological mechanism of delirium is far from clear [7]. Apart from other possible pathways related to the development of delirium [8], reduced cerebral blood flow during delirium with normalization during recovery has been reported [9, 10]. Cerebral blood flow is strongly related to whole blood viscosity (WBV) [11]. Changes in blood viscosity occur post-cardiothoracic surgery [12]. As such, changes in blood viscosity could relate to occurrence of postoperative delirium and this could represent an important interventional target to prevent or treat delirium.

Blood viscosity is higher at low shear rates, e.g. in the microcirculation [13]. Therefore, an increased WBV leads to a larger reduction in microcirculatory blood flow compared to blood flow in larger blood vessels. Since cellular perfusion is dependent on microcirculatory flow [14] and blood viscosity affects microcirculatory flow, we hypothesized that changes in viscosity could be related to the development of delirium. The aim of our study was to investigate whether whole blood viscosity differs between cardiothoracic surgery patients with and without delirium.

## Methods

### Study design and patients

This is an exploratory, matched, case–control study carried out in the Radboud University Medical Center, Nijmegen, The Netherlands. Annually approximately 1000 cardiothoracic patients are operated in the RadboudUMC. This study was approved by the medical ethical committee of Arnhem-Nijmegen (study number 2012/297) which waived the need for informed consent.

The study population consisted of patients of 50 years or older after an elective cardiothoracic surgical on-pump procedure for coronary artery bypass grafting (CABG) or single heart valve surgery. For purpose of homogeneity of the total group, patients who underwent CABG combined with valve surgery were not included. Other exclusion criteria were the use of blood cardioplegia, since this is associated with the development of postoperative neurological events [15], preoperative use of heparin, since heparin could decrease the blood viscosity [16], extracorporeal circulation (ECC) time exceeding 120 min, because ECC time is associated with neurological injury [17], or inability to screen for delirium.

Patients developing delirium postoperatively were defined as cases, and patients in which no delirium occurred served as non-cases. The group of cases was matched 1 : 4 to controls to increase the power of the study. Matching was performed on several important preoperative and postoperative risk factors for the development of delirium [7]: gender, age, duration of surgery, aortic cross clamp (AOX) time, ECC time, severity of illness score (Acute Physiology and Chronic Health Evaluation (APACHE)-II score), and risk of death after a heart operation (European System for Cardiac Operative Risk Evaluation (Euro score)).

### Delirium screening

Delirium assessment was performed three times a day. In order to obtain maximal sensitivity and specificity we used a three way approach to diagnose delirium. Firstly, in the ICU the most specific and sensitive scoring test for delirium, the validated Confusion Assessment Method for the ICU (CAM-ICU), was used by trained ICU nurses [18]. Secondly, for the non-ICU patients, on the cardiothoracic surgical ward, nurses used the validated Delirium Screening Observation (DOS) scale [19]. Unfortunately some patients are not diagnosed by validated tests [20]. Therefore, to not miss these patients nursing and medical files were screened for signs of delirium as the last approach [21]. Delirium was defined as having a positive CAM-ICU score, or a DOS scale ≥3. In order to maximize the sensitivity of the diagnosis delirium, we also checked the medical records when haloperidol was administered for treatment combined with delirium signs noted in the nursing or medical files in case of a negative CAM-ICU score.

### Data collection and variables

Earlier studies show that changes in blood viscosity occurs immediately after induction of anesthesia, immediately after surgery, 1 and 2 days after surgery and normalizes between 3 and 4 days post-cardiothoracic surgery [12]. For this reason blood samples were collected at four time points: preoperatively, directly after the induction of anesthesia (T_-1_), within one hour of ICU admission (T_0_), day one (T_24_) and three days (T_72_) after cardiothoracic surgery. Blood was drawn from the central venous catheter. If this was not possible, blood was taken from an indwelling arterial catheter or by vena puncture. During each blood collection the most important determinants of WBV, hematocrit and serum fibrinogen, were also measured and taken into consideration during viscosity calculations [11, 22]. Also presence of diabetes mellitus, infection confirmed by appropriate culture, invasive mechanical ventilation and the mean of the following variables during postoperative ICU stay in both groups were registered: serum creatinine level, modification of diet in renal disease-glomerular filtration rate (MDRD-GFR), urea level, fluid balance, ejection fraction (EF), mean arterial blood pressure (MAP), infusion rate of inotropes or vasopressors, partial thromboplastin time (PTT), glucose level, and temperature.

### Whole blood viscosity measurement

WBV is the intrinsic resistance of blood as it flows through blood vessels and is mainly determined by the shear rate of the flow, the volume fraction of red blood cells (hematocrit (Hct)), the concentration of plasma proteins namely fibrinogen, red blood cell (RBC) aggregation and red cell deformation [10, 23]. Viscosity can be represented as a function between shear rate and shear stress. Shear rate indicates the velocity of the blood flow and shear stress is the force of blood against the vessel wall. Fibrinogen has a greater influence on whole blood viscosity at low shear rates than at high shear rates due to fibrinogen induced RBC aggregation at low shear rates [24]. The interaction of fibrinogen and hematocrit on viscosity can be represented by an estimate of yield shear stress (YSS). YSS is the force required to start movement in a blood vessel [11, 22]. Furthermore, blood viscosity is dependent on temperature, especially at a temperature below 35 °C and above 39 °C [25, 26].

WBV was measured using the Contraves LS300 Low Shear Viscometer (ProRheo, Germany) within 180 min after blood sampling. The setting of the viscometer was standardized for all samples . Briefly, all blood tubes were placed on a shaker in the time between blood collection and the viscosity measurement. The viscosity was measured at 37 ± 0.1 °C, and at 23 different clinically relevant shear rate intervals (0.02-50 s^-1^) to minimize measurement errors [24]. As a measure for WBV, the area under the viscosity-shear rate curve (AUC) was used. The AUCs’ between the shear rates 0.02 and 5 s^-1^ were adjusted for Hct since this has a major impact on blood viscosity at low shear rates [11, 24]. Adjustment was performed by dividing the blood viscosity between the shear rates 0.02 and 5 s^-1^ by Hct. This is an estimation of adjusted whole blood viscosity for Hct approximating the precise value [27]. In addition, the yield shear stress was analyzed to compare the influence of Hct and fibrinogen on WBV. YSS was calculated according to Equation 1 [11, 22].

1 $$ \mathrm{Y}\mathrm{S}\mathrm{S}=13.5\left({10}^{-6}\right){C}_f^2{\left(Hct-6\right)}^3 $$

Where C_f_ is the fibrinogen concentration in mg%.

### Statistical analysis

We used a case:control ratio of 1:4, which resulted in a power of 94 %, with a two tailed alpha of 0.05. Student’s t-tests or Mann–Whitney U tests were used depending on data distribution. The Chi-square test was used to test the dichotomous variables. Because of the high level of attrition Linear Mixed Model testing was used to study the association between blood viscosity, hematocrit, fibrinogen and delirium. A two tailed *p* value of <0.05 was considered statistically significant. Statistical analysis was performed using IBM SPSS Statistics 20 and GraphPad Prism 5.0 (Graphpad Software, San Diego, CA, USA).

## Results

In total 80 cardiothoracic surgical patients were screened. Of these, 16 (20 %) developed delirium postoperatively. One non-delirious patient was excluded due to serious complications and sustained coma, five non-delirious and four delirious patients were excluded because of missing data. Subsequently 12 cases were matched with 48 non-cases. Nine patients developed delirium within 24 h, and 3 patients developed delirium within 72 h after surgery. Patient and demographic characteristics are depicted in Table 1.

**Table 1:** Demographic variables of delirious and non-delirious patients

Characteristic	Delirious (*N* = 12)	Non-delirious (*N* = 48)	*p*-value
Age (years), (mean, SD)	69 (5.6)	65 (8.2)	0.16
Male, N (%)	8 (67 %)	36 (75 %)	0.56
Duration of surgery in minutes (mean, SD)	195 (73)	187 (50)	0.65
ECC-time (min), (mean, SD)	107 (43)	90 (21)	0.21
AOX-time (min), (mean, SD)	65 (22)	59.8 (17)	0.36
APACHE-II Score (mean, SD)	17 (4)	15 (4)	0.19
Euro score (mean, SD)	3.8 (2.0)	3.5 (2.0)	0.68
Serum creatinine level (μmol. L-1), (median, IQR)	87 (68–94)	81 (73–94)	0.72
MDRD-GFR (ml. min-1. 1.73 m-2), (median, IQR)	78 (59–90)	81 (64–90)	0.49
Urea level (mmol. L-1), (median, IQR)	8.0 (7.2–9.1)	7.3 (6.0–8.6)	0.20
Fluid balance (mL), (median, IQR)	909 (34–1171)	375 (-137–1121)	0.65
EF (%), (median, IQR)	60.0 (46.3–60.0)	55.0 (45.0–60.0)	0.40
MAP (mmHg), (median, IQR)	75 (68–77)	75 (71–83)	0.40
Admission of inotropes or vasopressors, N (%)	10 (83 %)	28 (58 %)	0.11
Duration of mechanical ventilation (≥24 h), N (%)	0 (0 %)	0 (0 %)	N/A
PTT (sec), (median, IQR)	19 (18–21)	18 (18–19)	0.26
Diabetes mellitus, N (%)	0 (0 %)	5 (10 %)	0.83
Glucose level (mmol. L-1), (median, IQR)	7.9 (7.3–8.8)	7.6 (6.7–8.2)	0.36
Temperature (°C), (median, IQR)	36.6 (35.3–37.1)	36.6 (35.9–36.9)	0.93
Confirmed infection, N (%)	0 (0 %)	0 (0 %)	N/A

Definition of abbreviations: *ECC* extracorporeal circulation, *AOX* aortic cross clamp, *min* minutes, *APACHE II* acute physiology and chronic health evaluation II, *Euro* European system for cardiac operative risk evaluation, *IQR* interquartile range, *MDRD-GFR* modification of diet in renal disease-glomerular filtration rate, *EF* ejection fraction, *MAP* mean arterial blood pressure, *N/A* not applicable, *PTT* partial thromboplastin time, *sec* seconds, *SD* standard deviation

### Postoperative levels of fibrinogen, hematocrit and whole blood viscosity

**Fig. 1:**
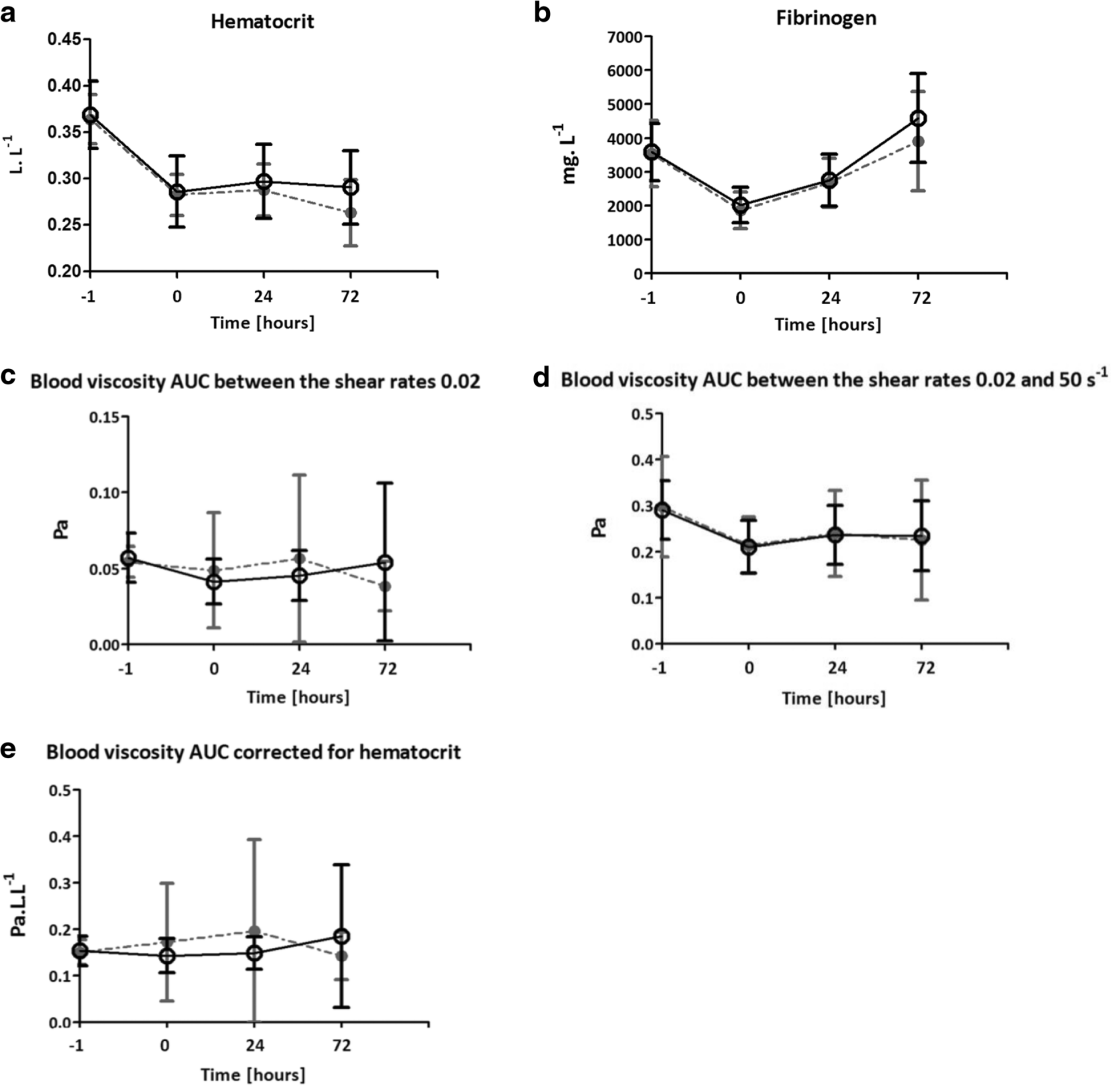
Postoperative levels of hematocrit, fibrinogen and whole blood viscosity of delirious and non-delirious patients. **a** Hematocrit. **b** Fibrinogen. **c** The area under curve (AUC) of the whole blood viscosity (WBV) between shear rates 0.02 and 5 s^-1^. **d** The area under curve (AUC) of the whole blood viscosity (WBV) between shear rates 0.02 and 50 s^-1^. **e** The area under curve (AUC) of the whole blood viscosity corrected for hematocrit. T_-1_, directly after the induction of anesthesia. T_0_, within one hour of Intensive Care Unit admission. T_24_, one day after cardiothoracic surgery. T_72_, three days after cardiothoracic surgery. Data are expressed as mean and SD. Linear Mixed Model testing was used to determine a difference between the two groups. No significant differences were found. A two tailed p value of < 0.05 was considered statistically significant

**Fig. 2:**
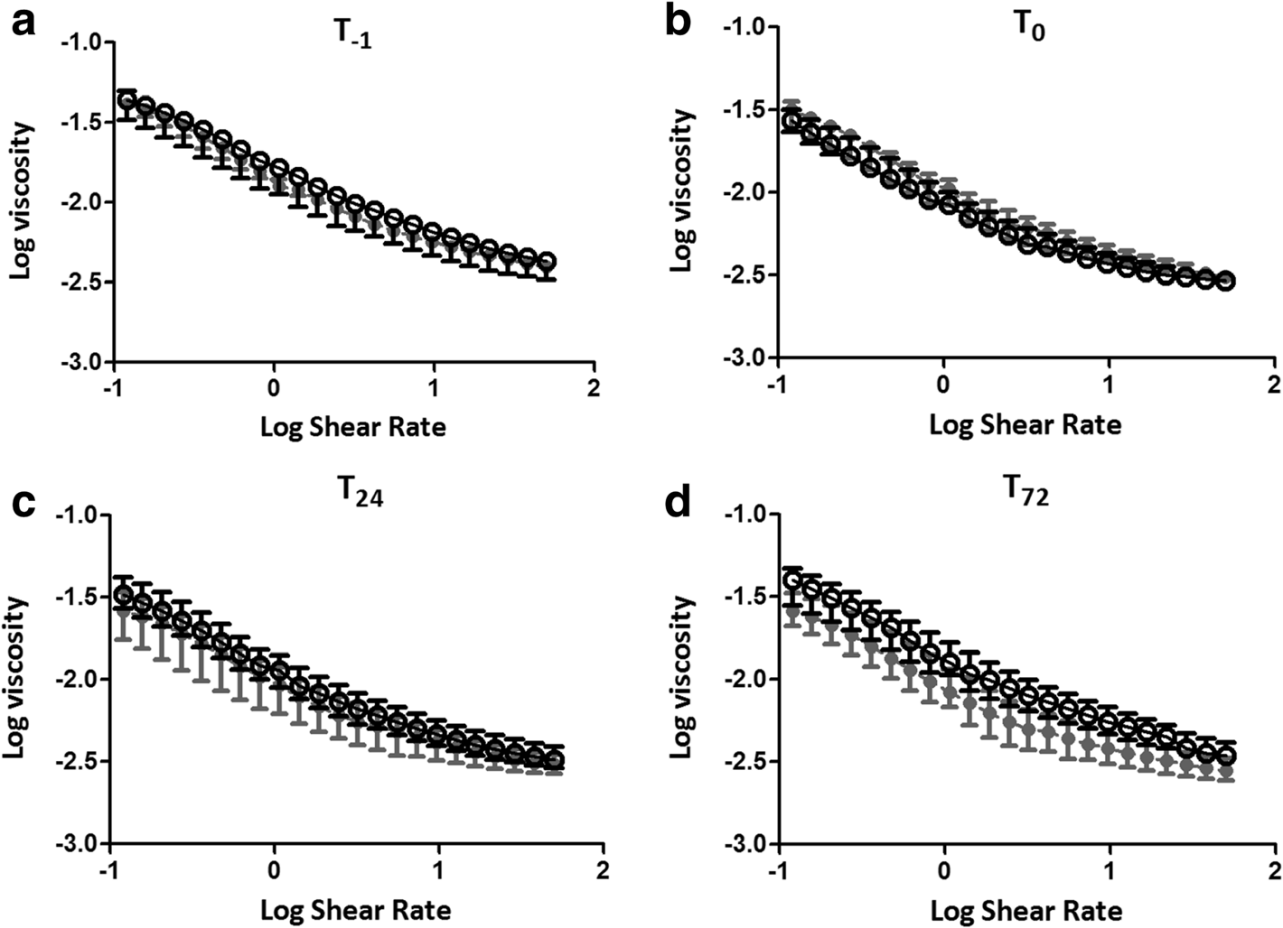
Whole blood viscosity at 23 different shear rates between 0.02 and 50 s^-1^ of delirious and non-delirious patients. Log10 of whole blood viscosity versus log10 of shear rates. **a** Directly after the induction of anesthesia (T_-1_). **b** Within one hour of Intensive Care Unit admission (T_0_). **C** One day after cardiothoracic surgery (T_24_). **d** Three days after cardiothoracic surgery (T_72_)

Pre-operative fibrinogen, Hct and WBV were comparable between groups (Fig. 1). In both groups fibrinogen levels and hematocrit decreased significantly after surgery (both *p* <0.001). No significant difference was found between both groups in reduction of fibrinogen (*p* = 0.36) and hematocrit (*p* = 0.23), Fig. 1. Postoperatively the AUC of WBV between shear rates 0.02 and 50 s^-1^ decreased significantly in both groups (*p < 0.001).* Again, there was no significant difference in this reduction between patients that developed delirium and those who did not (*p* = 0.80). The AUC of WBV between shear rates 0.02 and 5 s^-1^ remained similar over time in both groups and not different between both groups (*p* = 0.78) either. The AUC of blood viscosity adjusted for hematocrit was also comparable between the patients who developed delirium and those who did not (*p* = 0.33), (Figs. 1and 2). Finally, changes in the YSS were also not different between both groups (*p* = 0.68).

Changes in the Hct, fibrinogen, WBV and YSS did not differ significantly in patients that developed delirium within 24 h and those that developed delirium after 48 h.

## Discussion

We hypothesized that development of delirium in post-cardiothoracic surgery patients could be related to changes in blood viscosity. In line with such a hypothesis is the fact that reduced cerebral blood flow has been suggested to be a possible pathway for the occurrence of delirium. In addition, strokes and dementia are correlated with occurrence of delirium. Both strokes and dementia have been associated with high blood viscosity [28, 29] and both occur more frequently after cardiothoracic surgery. In this exploratory case–control study we did not find differences in the changes in hematocrit, fibrinogen levels or whole blood viscosity in post-cardiothoracic surgery patients who did or did not develop delirium. These findings indicate that changes in viscosity during the postoperative phase do not play a role in the development of post-operative delirium in cardiothoracic surgery patients. Even more so, the observed postoperative decrease of Hct and WBV provides no protection against delirium.

Although several studies have shown a strong correlation between whole blood viscosity and cerebral flow [11, 24], it appears plausible that due to low hematocrit levels after cardiothoracic surgery, the viscosity level is already so low, that moderate changes at this low level of blood viscosity do not affect cerebral blood flow. It has been shown, that a logarithmic correlation exists between hematocrit and whole blood viscosity, which is even stronger at lower shear rates [30]. Another reason why our hypothesis was not confirmed could be, that cerebral blood flow and delirium are not strongly correlated. However, earlier studies have demonstrated a correlation between decreased cerebral blood flow and delirium [9, 10].

The postoperative changes in WBV we observed are in accordance with previous reports in cardiothoracic surgery patients [12, 31]. However, in those studies, viscocity was only measured at a high shear rate (90 s^-1^), while in our study we measured viscosity at 23 different and at low shear rates. We found no postoperative changes in WBV at shear rates between 0.02 and 5 s^-1^ in cardiothoracic surgery patients. At low shear rates, red blood cells clump together due to fibrinogen induced RBC aggregation resulting in a higher viscosity which is dependent on both Hct and fibrinogen concentration [11, 24]. Therefore, unlike Papp et al. [12], we took a small part of the blood viscosity curve at low shear rates (between 0.02 and 5 s^-1^) and corrected for hematocrit. Nevertheless, there was no significant difference between delirious and non-delirious patients.

Although duration of delirium is not a true reflection of severity of delirium, this is in several studies used as a measure for delirium severity. However, in our study the maximum delirium duration was 3 days (1 patient) and the rest had 1–2 days of delirium. For this reason we have treated delirium as a dichotomous variable. Data from other studies show that the median duration of delirium in cardiac surgery patients is two days [3, 32]. This is comparable to our data.

Some limitations of this study need to be considered. Firstly, the gold standard to diagnose delirium was not used. The gold standard is clinical research, performed by a psychiatrist, a neuropsychologist or a geriatrician. Delirium fluctuates during the day, therefore the gold standard is not always practical [33]. Instead of the gold standard, the internationally validated CAM-ICU and DOS scale performed by nurses were used to enable multiple assessments per day per patient [19]. At each nursing shift, patients were screened for delirium. Using multiple assessments per day increases the sensitivity of delirium diagnosis. In addition, in order to minimize under-diagnosis, the reports of the doctors and nurses were analyzed for indications of delirium in combination with the use of anti-psychotics. Secondly, in this study we included both the CABG as well as the aortic valve surgery patients. In comparison to closed heart surgery, patients undergoing open heart surgery have an increased risk for cerebral embolization [34, 35]. Although, the latter introduces heterogeneity, it results in data that can be generalized more easily to the daily practice of cardiac surgery. Finally, in this study 25 % of the data is missing. However, we used Linear Mixed Models testing in which it is allowed to have 25–30 % of missing [36].

## Conclusion

In this group of cardiothoracic surgery patients no association was found between whole blood viscosity and the development of post-operative delirium. This finding indicates that in postoperative cardiothoracic surgery patients, delirium is probably not related to blood viscosity changes.

## Abbreviations

AOX, aortic cross clamp; APACHE, acute physiology and chronic health evaluation; AUC, area under the curve; CABG, coronary artery bypass grafting; CAM-ICU, confusion assessment method intensive care unit; C_f_, fibrinogen concentration; DOS, delirium screening observation; ECC, extracorporeal circulation; EF, ejection fraction; Euro score, European system for cardiac operative risk evaluation; Hct, hematocrit; ICU, intensive care unit; IQR, interquartile range; MAP, mean arterial blood pressure; MDRD-GFR, modification of diet in renal disease-glomerular filtration rate; N/A, not applicable; PTT, partial thromboplastin time; RBC, red blood cell; SD, standard deviation; WBV, whole blood viscosity; YSS, yield shear stress.
